# Hyperbaric Oxygen Therapy for Necrotizing Soft Tissue Infections: A Retrospective Cohort Analysis of Clinical Outcomes

**DOI:** 10.1089/sur.2024.285

**Published:** 2025-07-30

**Authors:** Akira Shishido, Gregory Schrank, Alexander Vostal, Megan Uehling, Ravi Tripathi, Sai Chintalapati, Lauren Conway, Nikki Kus, Laura DiChiacchio, Marc Kai, Joseph A. Kufera, Ronald Rabinowitz

**Affiliations:** ^1^ University of Maryland School of Medicine, Baltimore, Maryland, USA.; ^2^ Laboratory of Infectious Diseases, National Institute of Allergy and Infectious Diseases, National Institutes of Health, Bethesda, Maryland, USA.; ^3^ National Study Center for Trauma and Emergency Medical Systems, Center for Shock, Trauma and Anesthesiology Research, Baltimore, Maryland, USA.

**Keywords:** hyperbaric oxygen therapy, necrotizing fasciitis

## Abstract

**Background:**

Hyperbaric oxygen therapy (HBOT) is an adjunctive therapy for necrotizing soft tissue infections (NSTIs) that remains controversial due to lack of quality clinical evidence. This retrospective cohort examines the impact of HBOT on clinical outcomes from NSTI at a single center where evaluation for HBOT is standard of care.

**Methods:**

The COVID-19 pandemic disrupted HBOT service and NSTI cases went without HBOT treatment, allowing for a comparison of treatment groups. The clinical outcomes of 253 patients with NSTI that were evaluated for HBOT were compared; 143 (56.3%) received HBOT and 110 (43.3%) did not.

**Results:**

Baseline characteristics were similar except for surface area of the wounds and distribution on the extremities. More patients in the non-HBOT group died within 90 days of admission than those in the HBOT group (5.8% vs. 15.4%, p = 0.015). Further, patients with large wounds (≥450 cm^2^) and those with high APACHE II scores (≥18) who underwent HBOT had significantly lower risk of death than patients who did not (odds ratio [OR] 0.12, 95% confidence interval [CI] 0.02–0.72).

**Conclusion:**

Our study shows that there was a mortality benefit in patients with NSTI that was more significant in patients with large wounds and higher APACHE II scores.

## Introduction

Necrotizing soft tissue infections (NSTIs) are a group of surgical diagnoses characterized by wide-spread tissue destruction from either a mono- or polymicrobial infection.^1^ NSTIs remain a significant cause of morbidity and mortality; even with optimal treatment regimens of today, mortality rates range as high as 25%–35%.^2^ Current treatment standards include immediate surgical intervention with aggressive debridement of necrotic tissue and broad-spectrum antibiotic agents accompanied by hemodynamic and respiratory support. One adjunctive therapy not universally used includes the use of hyperbaric oxygen therapy (HBOT). During HBOT, the partial pressure of oxygen in body tissue and wounds is increased beyond what would be physiologically possible at ambient atmospheric pressure. This hyperoxia is proposed to prevent ischemia and necrosis in poorly perfused infected tissue and produces reactive oxygen species (ROS) intensifying the immune response to infection.^3^ In addition, HBOT itself is associated with antibacterial affects.^3,4^ Despite the theoretical benefits of HBOT, its use remains controversial given the significant investment of health care resources required and the lack of quality clinical evidence.

The R Adams Cowley Shock Trauma Center at the University of Maryland Medical Center (UMMC) is a regional referral center for the treatment of NSTI, with a dedicated soft tissue surgical service and hyperbaric medicine team available 24/7 for emergent procedures. As part of local practice, all patients admitted to UMMC with a diagnosis of NSTI and undergo surgical debridement are evaluated for adjuvant HBOT. The dose and duration of HBOT are determined by the treating hyperbaric medicine physician in coordination with the surgical team based upon their operative findings.^5^ Typically, the treatment protocol for NSTI includes initiating HBOT at 2.0 to 2.5 ATA pressure for 90 min of oxygen given twice a day for the first few days, until there appears to be no further extension of necrosis in previously debrided areas and infection is controlled.^6^ During the first few months of the COVID-19 pandemic, significant changes to the allocation of hospital resources to care for COVID-19 patient surges, reduced patient volumes for conditions other than COVID-19, and infection control concerns related to the potential spread of respiratory infections within the HBOT treatment area significantly decreased the utilization of this treatment for NSTI. The abrupt change in the management of NSTI without routine use of adjuvant HBOT provided an opportunity to explore any association between the change in practice and patient outcomes. Our study aim was to assess the association between mortality and the receipt of HBOT as adjuvant treatment for NSTI. We hypothesized that among patients with NSTI, receipt of HBOT would be associated with a lower risk of mortality in comparison with those who did not receive HBOT.

## Methods

We conducted a retrospective cohort study of patients admitted to our 800-bed tertiary-care academic medical center in Baltimore, Maryland. The University of Maryland, Baltimore Institutional Review Board reviewed the study protocol and determined it to be exempt. Patients were included if they were aged ≥18 years with a diagnosis of NSTI between January 2018 and December 2020. Each admitted patient with a diagnosis of NSTI as determined by the primary surgical service receives a consultation from hyperbaric medicine for consideration of HBOT and is considered eligible for treatment if having an active necrotizing soft tissue that has undergone surgical debridement. Contraindications to HBOT include untreated pneumothoraces, agitation or behavioral challenges, and severe hemodynamic instability as determined by hyperbaric medicine and intensive care teams on a case-by-case basis. Patients were excluded if limb amputation or death occurred within 48 h of admission.

On March 20, 2020, the HBOT chamber ceased normal operations and patients with NSTI were no longer routinely referred for consultation. HBOT operations were partially resumed beginning June 2, 2020, with gradual increases in patient volumes over time, preferentially focused on inpatient consultations, including NSTIs. This unexpected closure of the HBOT chamber created an increase in the number of patients with NSTI who would have normally received HBOT. Data collected included patient demographics, baseline comorbidities, wound characteristics, microbiology, APACHE II score upon admission, and receipt of HBOT. The primary outcome was 90-day mortality. Secondary outcomes were limb amputation, inpatient antibiotic agent days, ventilator days, and hospital length of stay.

Bivariate comparisons between the HBOT and non-HBOT groups were made using Pearson’s chi-square statistic and Fisher exact test for categorical variables. For continuous variables, comparisons were made with the Student’s *t* test for normally distributed data and the Mann–Whitney test for asymmetrical variables. Multivariable logistic regression models were constructed to determine the effect of HBOT, modified by wound size and severity of illness in the form of interaction terms, on the outcome of 90-day mortality. A post hoc subgroup analysis of patients with APACHE-II scores and wound size greater than the median cohort value was performed using three-way interaction terms to examine the effect of HBOT on 90-day mortality using logistic regression. With the small number of death outcomes, we were limited in the number of variables that could be included in the regression analysis. We selected variables that were clinically relevant with a plausibility of being predictors of the outcome of interest and those with differences between groups. For all analyses, a p value <0.05 was considered statistically significant. Analysis was performed using SAS® software, version 9.4 (SAS Institute, Cary, NC).

The authors completed the STROBE checklist before submitting this article.

## Results

**FIG. 1.**
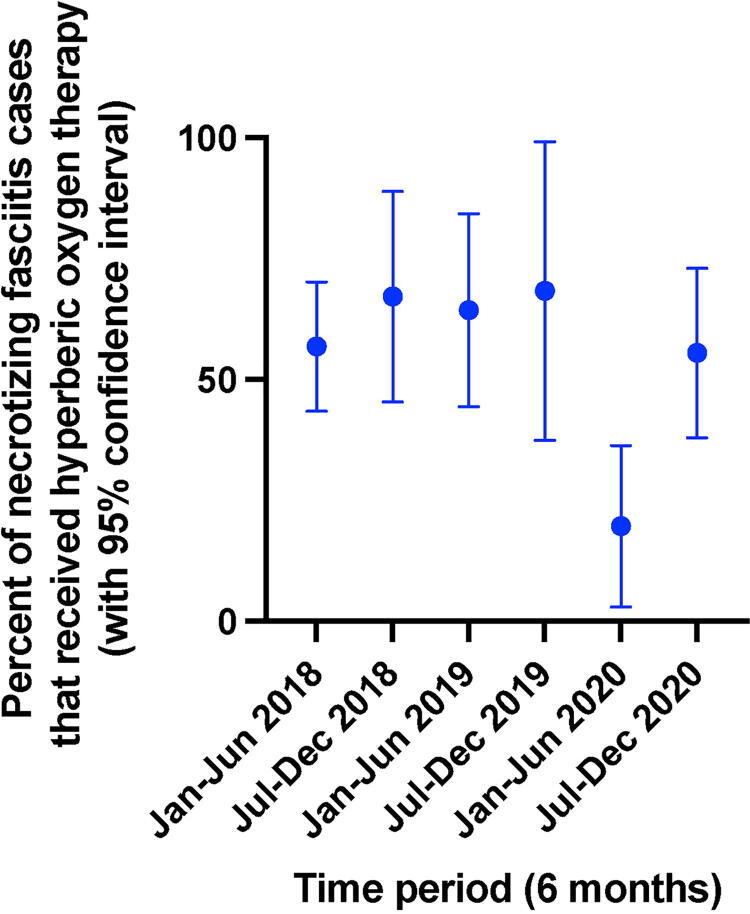
Percentage of necrotizing soft tissue infection (NSTI) patients admitted to R Adams Cowley Shock Trauma Center who received hyperbaric oxygen therapy (HBOT) from January 2018 through December 2020.

During the study period, 253 patients met inclusion criteria, of whom 143 (56.3%) received HBOT and 110 (43.3%) did not. The percentage of patients who underwent HBOT dropped from 68% to 20% during the first six months of the COVID-19 pandemic (Fig. 1). Baseline characteristics were similar between groups (Table 1). Most patients were male and the mean age was approximately 53 years in both groups. Over 90% of the patients were transferred to our facility. There were no statistically significant differences in patient comorbidities. Nearly half of the patients had a pre-existing diagnosis of diabetes mellitus and close to 20% of the patients had substance abuse disorders. Fewer than 10% of patients were immunosuppressed, but this was similar in both groups. The number of surgical debridement procedures performed at an outside facility before transfer (if applicable), or causative organisms, was similar as well. Nearly 60% of the infections involved the perineal area. Median APACHE II score was higher in patients who did not undergo HBOT but did not meet statistical significance. The only differences in baseline characteristics between the two groups were wound surface area, which was greater in the HBOT group (median 525 cm^2^ vs. 304 cm^2^, p = 0.005), and the anatomical location of infection was more likely to be on an extremity in the HBOT group (43.2% vs. 27.5%, p = 0.016).

**Table 1.** Comparison of Patient Demographics in Hyperbaric Oxygen Therapy Versus No Hyperbaric Oxygen Therapy Groups

Variable	HBOT no. (%)	No HBOT no. (%)	*p* Value
Patients	143 (56.3%)	110 (43.3%)	
Total			
Male	87 (62.6%)	49 (53.8%)	0.187^*^
Age, mean ± SD	53.5 ± 13.9	52.2 ± 15.5	0.494^#^
Transferred from OSH	129 (92.8%)	82 (90.1%)	0.468^*^
Number of Debridements at OSH			
0	80 (66.1%)	45 (66.2%)	
1	27 (22.3%)	18 (26.5%)	
> = 2	14 (11.6%)	5 (7.4%)	0.581^*^
Comorbidities			
Diabetes	78 (56.1%)	43 (47.3%)	0.188^*^
Chronic kidney disease	17 (12.2%)	13 (14.3%)	0.651^*^
Peripheral vascular disease	8 (5.8%)	1 (1.1%)	0.091^**^
Substance abuse	23 (16.5%)	17 (18.7%)	0.676^*^
Smoker	48 (34.5%)	25 (27.5%)	0.261^*^
BMI, median (Q1, Q3)	32.0 (25.8,40.1)	33.0 (25.7,43.1)	0.344^##^
Chronic steroids	9 (6.5%)	3 (3.3%)	0.372^**^
Uncontrolled HIV	1 (0.7%)	1 (1.1%)	—
SOT	2 (1.4%)	1 (1.1%)	—
HSCT	0 (0%)	0 (0%)	—
Chemotherapy	2 (1.4%)	3 (3.3%)	0.387^**^
Body Location of Infection			
Head and neck	2 (1.4%)	1 (1.1%)	—
Perineal	83 (59.7%)	52 (57.1%)	0.699^*^
Abdominal	15 (10.8%)	10 (11.0%)	0.962^*^
Truncal	8 (5.8%)	9 (9.9%)	0.241^*^
Extremity	60 (43.2%)	25 (27.5%)	0.016^*^
Inguinal	14 (10.1%)	9 (9.9%)	0.964^*^
Time to 1st operation, (h) median (Q1, Q3)	5.0 (3.0,9.0)	5.0 (3.0,10.0)	0.843^##^
Total surface area, (cm^2^) Median (Q1, Q3)	525.0 (255.0,828.0)	304.0 (173.3,643.8)	0.005^##^
APACHE II, median (Q1, Q3)	12.0 (7.0,16.3)	14.0 (7.3,21.0)	0.067^##^
Microbiology			
Beta hemolytic Strep	45 (32.4%)	19 (20.9%)	0.057^**^
Clostridial species	2 (1.4%)	4 (4.4%)	0.217^*^
MSSA	11 (7.9%)	3 (3.3%)	0.152^*^
MRSA	5 (3.6%)	7 (7.7%)	0.227^**^
Gram-negative bacilli	47 (33.8%)	31 (34.1%)	0.968^*^
Polymicrobial	10 (7.2%)	6 (6.6%)	0.861^*^
Other	49 (35.3%)	43 (47.3%)	0.069^*^

^*^ p Value using chi-square test.

^**^ p Value using Fisher exact test.

^#^ p value using independent *t* test.

^##^ p Value using Mann–Whitney U test.

OSH = outside hospital; BMI = body mass index; HSCT = hematopoietic stem cell transplant; HIV = human immunodeficiency virus; MRSA = methicillin-resistant *Staphylococcus aureus*; MSSA = methicillin-sensitive *Staphylococcus aureus*; SOT = solid organ transplant.

Mortality at 90 days was lower in patients who received treatment with HBOT, compared with those who did not (5.8% vs. 15.4%, p = 0.015, Table 2). Length of stay and inpatient antibiotic days were longer in patients who received HBOT (15.1 vs. 11.7 d, p = 0.015, 13.0 vs. 11.0, p = 0.002). Rates of amputation were low, and not statistically different between the groups.

**Table 2.** Comparison of Outcomes Between Patients Who Received Hyperbaric Oxygen Therapy and Those Who Did Not

Outcome	HBOT no. (%)	No HBOT no. (%)	*p* Value
Died within 90 d of admission	8 (5.8%)	14 (15.4%)	0.015
Number of operations, median (Q1, Q3)	3.0 (2.0,4.0)	3.0 (2.0,4.0)	0.037
Inpatient antibiotic agent days, median (Q1, Q3)	13.0 (9.0,16.0)	11.0 (7.0,15.0)	0.002
Ventilator days, median (Q1, Q3)	1.0 (0.0,4.0)	0.0 (0.0,4.0)	0.65
Length of stay, median (Q1, Q3)	15.1 (9.8,22.2)	11.7 (7.0,21.2)	0.015
Amputation performed	7 (5.0%)	2 (2.2%)	0.489

We identified median values for APACHE II scores and wound size and used them as the lower limit cutoffs for post hoc analysis. In post hoc subgroup analysis of mortality, patients with APACHE II scores ≥18 who underwent HBOT had significantly lower risk of death than patients in the non-HBOT group (odds ratio [OR] 0.23, 95% confidence interval [CI] 0.06–0.95). Among patients with wounds ≥450 cm^2^, those who underwent HBOT had significantly lower risk of death than patients in the non-HBOT group (OR 0.22, 95% CI 0.07–0.70). Patients with both APACHE II scores ≥18 and wounds ≥450 cm^2^ who underwent HBOT had an even lower risk of death than patients in the non-HBOT group (OR 0.12, 95% CI 0.02–0.72, Table 3). There was no statistically significant difference in mortality between HBOT treatment groups for patients with APACHE II scores <18 or wound sizes <450 cm^2^.

**Table 3.** Logistic Regression of Effect of Hyperbaric Oxygen Therapy on Death Within 90 Days, Comparing Interactions of HBOT with APACHE II Score and Wound Size

Post hoc subgroup analysis of death within 90 days	Odds ratio	95% Confidence limits		*p* Value
Effect of HBOT among APACHE II <18	0.88	0.20	3.83	0.87
Effect of HBOT among APACHE ≥ 18	0.23	0.06	0.95	0.04
Effect of HBOT among wound size < 450	0.88	0.12	6.51	0.90
Effect of HBOT among wound size ≥ 450	0.22	0.07	0.70	0.01
Effect of HBOT among APACHE < 18 and wound size < 450	5.37	0.18	159.40	0.33
Effect of HBOT among APACHE < 18 and wound size ≥ 450	1.36	0.14	13.34	0.79
Effect of HBOT among APACHE ≥ 18 and wound size < 450	1.20	0.06	22.30	0.90
Effect of HBOT among APACHE ≥ 18 and wound size ≥ 450	0.12	0.02	0.72	0.02

## Discussion

Our retrospective study compared the outcomes of patients with NSTI who did or did not receive HBOT and found that receipt of HBOT was associated with lower 90-day mortality, with the greatest benefit in patients with higher APACHE II scores on admission and large wound sizes. Our data are consistent with previous studies that suggest a mortality benefit of HBOT in patients with NSTIs.^7–12^

The largest recent studies are retrospective in nature and suffer from inherent selection and treatment biases as well as heterogeneity. Soh et al. performed a very large database analysis in 2012 reviewing ICD-9 data across the United States.^8^ The study pooled data from 45,913 patients diagnosed with NSTIs over a 20-year period. In total, 405 patients received HBOT, while the others did not. Mortality was significantly lower in the HBOT group (12% vs. 24%); however, the HBOT group had less severe initial illness.^8^ In addition, the data came from multiple centers, many of which did not offer HBOT. Shaw et al. attempted to correct for the issue of HBOT accessibility by pooling data only from centers that offered HBOT.^9^ Over a two-year period 1,583 patients with NSTI were identified, of which 7% were treated with HBOT. The authors found no difference in hospital length of stay, cost, and mortality in less severe cases on the basis of the severity of illness (SOI) score; but they identified a lower complication rate (45% vs. 66%) and lower mortality rate (4% vs. 23%) in patients with most severe illness, a finding consistent with our results.^9^ Devaney et al. performed a single study retrospective cohort study in Australia covering a 13-year period and also found an overall mortality benefit (14% vs. 24%) among a total of 341 patients.^13^

In a more recent meta-analysis performed by Hedetoft et al. published in 2021, over 500 studies were initially identified, but only 21 studies met their major inclusion criteria for in-hospital mortality and a secondary outcome of major amputation.^7^ This review encompassed 48,744 total patients, of which 1,237 received HBOT. Consistent with the findings of our study, the odds of mortality were lower in patients who received HBOT with an OR of 0.44. In a smaller number of the studies, the overall OR for major amputations was 0.6, favoring HBOT.^7^

Huang et al. published a large systematic review in 2023 that showed a significantly lower mortality in patients undergoing HBOT (RR = 0.522).^12^ However, the data were significantly heterogenous and the number of debridement procedures were higher in the HBOT group.^12^ Lastly, Toppen et. al analyzed a large inpatient database between 2012 and 2020 for associations with HBOT and mortality, amputations, length of stay, and cost.^11^ After correcting for differences, they found that HBOT was significantly associated with decreased mortality and amputations but with increased costs and lengths of stay as would be expected in cases of longer survival.^11^

Our study is unique in that it is the only recent study that specifically associated the benefit of HBOT with the size of the wounds. Given that HBOT has many physiological effects that can promote wound healing and increase oxygenation, it is not surprising that larger wounds would benefit from HBOT as they are often more devitalized. While it is possible that patients with larger wounds were more likely to be selected for HBOT, a large wound size is not a considered criterion for HBOT at our institution. It is possible that patients with large wounds in the non-HBOT group had undergone complete amputation before evaluation for HBOT rendering the modality; however, we attempted to control for this by excluding all patients who underwent an amputation within 48 h of presentation.

In our post hoc subgroup analysis, higher severity of illness as measured by APAHE II score was associated with a lower mortality with HBOT. Although APACHE II is not an all-encompassing measure of illness severity (i.e., it does not include pressor requirements), we do feel it is a reliable standard for comparison and it is impressive that ostensibly sicker patients had improved mortality with HBOT, as also noted in Shaw et al.^9^

The strengths of our study are that it was performed at a regional referral center for NSTI, with high volumes of referred cases and where HBOT is standard of care for all patients with NSTI who undergo debridement, resulting in a large single-center cohort. This is particularly notable given the relatively low incidence of NSTI and lack of HBOT availability in general, making direct comparison of treatment strategies challenging for most facilities. One limitation of this study is that due to the observational nature of the analysis, it is possible that other facets of care also changed during the COVID-19 pandemic that may have contributed to patient outcomes negatively, including the increase in hospital-acquired infections and the increase of multiple drug-resistant organisms.^14^ Further, inherent to this study being a retrospective analysis on the basis of effects of the COVID-19 pandemic, and not a randomized controlled trial, there is likely an element of selection bias affecting which patients were selected for HBOT and which were not. We attempted to account for this bias when selecting our variables for comparison. In addition, while our data suggest that patients who received HBOT experienced longer hospital stays and antibiotic agent durations, this result as well as other secondary outcomes may be directly related to the mortality benefit observed in the HBOT group.

The use of APACHE II score in our study was calculated on the basis of the first set of values at our institution, not those from the referring institutions for those patients who were referred. It is therefore possible that the initial severity of illness was not accurately represented. Other limitations of this study include the small overall number of patients included in analysis, the low mortality rate of the cohort overall, and omission of the specific HBOT “dose” (i.e., duration and pressure) for all patients who received it. Lastly, our 90-day mortality was limited to data available within the electronic medical record shared across several regional health systems; therefore, it is possible that some patients may have died without our knowledge.

## Conclusion

In this population of patients with NSTI who underwent surgical debridement, HBOT was associated with lower 90-day mortality. The difference in mortality appears most profound in patients who had large wounds (≥450 cm^2^) and severe illness (APACHE II score ≥18). A prospective study should further evaluate the impact of HBOT on mortality.

## Data Availability

Data are available upon request.
